# Characterization and Prediction of Protein Phosphorylation Hotspots in *Arabidopsis thaliana*

**DOI:** 10.3389/fpls.2012.00207

**Published:** 2012-09-05

**Authors:** Jan-Ole Christian, Rostyslav Braginets, Waltraud X. Schulze, Dirk Walther

**Affiliations:** ^1^Max Planck Institute for Molecular Plant PhysiologyPotsdam-Golm, Brandenburg, Germany; ^2^Bioinformatics, Universität PotsdamPotsdam-Golm, Germany

**Keywords:** protein phosphorylation, hotspots, *Arabidopsis thaliana*, support vector machines, regulation

## Abstract

The regulation of protein function by modulating the surface charge status via sequence-locally enriched phosphorylation sites (P-sites) in so called phosphorylation “hotspots” has gained increased attention in recent years. We set out to identify P-hotspots in the model plant *Arabidopsis thaliana*. We analyzed the spacing of experimentally detected P-sites within peptide-covered regions along *Arabidopsis* protein sequences as available from the PhosPhAt database. Confirming earlier reports (Schweiger and Linial, 2010), we found that, indeed, P-sites tend to cluster and that distributions between serine and threonine P-sites to their respected closest next P-site differ significantly from those for tyrosine P-sites. The ability to predict P-hotspots by applying available computational P-site prediction programs that focus on identifying single P-sites was observed to be severely compromised by the inevitable interference of nearby P-sites. We devised a new approach, named HotSPotter, for the prediction of phosphorylation hotspots. HotSPotter is based primarily on local amino acid compositional preferences rather than sequence position-specific motifs and uses support vector machines as the underlying classification engine. HotSPotter correctly identified experimentally determined phosphorylation hotspots in *A. thaliana* with high accuracy. Applied to the *Arabidopsis* proteome, HotSPotter-predicted 13,677 candidate P-hotspots in 9,599 proteins corresponding to 7,847 unique genes. Hotspot containing proteins are involved predominantly in signaling processes confirming the surmised modulating role of hotspots in signaling and interaction events. Our study provides new bioinformatics means to identify phosphorylation hotspots and lays the basis for further investigating novel candidate P-hotspots. All phosphorylation hotspot annotations and predictions have been made available as part of the PhosPhAt database at http://phosphat.mpimp-golm.mpg.de.

## Introduction

Protein phosphorylation is one of the most significant and best characterized posttranslational modifications involved a wide range of molecular regulatory and signaling mechanisms (Johnson, 2009). Conventionally, functionally relevant phosphorylation sites (P-sites) have been regarded as precisely defined sites within proteins. Their exact location along the protein sequence, and thus in the three-dimensional structure, was thought to be a key determinant for their exerted function, for example, by inducing conformational changes of the associated protein allowing for allosteric regulation (Barford et al., 1991). In agreement with this view, functionally relevant P-sites have been observed to be conserved in evolution such that their position in the sequence and structure of proteins is maintained across different bacteria (Macek et al., 2007), plants (Nakagami et al., 2010), vertebrates (Malik et al., 2008), and eukaryotes in general (Boekhorst et al., 2008). This conclusion has been challenged by other studies that reported that P-sites are no more conserved than expected by chance (e.g., in a comparison of mouse and human; Jimenez et al., 2007). In a recent survey of amino acid changing polymorphisms across many *Arabidopsis thaliana* accessions, it was concluded that serine, threonine, and tyrosine sites associated with phosphorylation events were indeed statistically more conserved than their non-phosphorylated counterparts. However, statistical significance was established at *p* = 0.03 only (Joshi et al., 2012). Given that several thousands of positions were included in the test, this weak significance is rather surprising.

An alternative view on the role of P-site localization and the consequences for protein function emerged when it was observed that, rather than individual sites with determined position, clusters of P-sites appear to be functionally significant, in which the exact location of individual P-sites belonging to the cluster seems less important (Moses et al., 2007). Instead, the combined effect of the individual sites on the local electrostatic surface potential appears to be relevant. For example, in an analysis of P-sites in cell-cycle dependent kinases (Cdk1) across several ascomycete species, it became evident that instead of the individual sites, a rather unspecific cluster of P-sites with evolutionarily rapidly changing positions of individual P-sites appeared to be conserved and thus functionally relevant for modifying protein–protein interactions (Holt et al., 2009). Similarly, the function of the protein Ste5, involved in the regulation of cell cycle in yeast in response to pheromones, was observed to be regulated via eight poorly conserved serine and threonine P-sites (Strickfaden et al., 2007). The regulatory switch was identified as a disruption of Ste5 binding to the inner leaflet of the cellular membrane caused by the negative charges transferred by phosphorylation. It has furthermore been argued that, if the exact location of a particular P-site is not conserved, the mechanism of phosphorylation-mediated signaling can still be preserved if alternative sites are phosphorylated by orthologous kinases in the respective organism (Tan et al., 2010). Consequently, effects mediated via the electrostatic surface potential alone rather than position-specific changes of protein structure were increasingly discussed as possible modes of action of phosphorylation-mediated regulation (Serber and Ferrell, 2007). For surface electrostatics to take effect, all that is required is a local accumulation of charges reflected by a tendency for P-sites to cluster along the sequence and structure of proteins. The precise location of P-sites would then be secondary in this respect. Indeed, such clusterings of P-sites, so called P-hotspots, have been observed for individual proteins, e.g., CDK targets (Moses et al., 2007) and on a broader proteomic scale in the plant *A. thaliana* (Riano-Pachon et al., 2010), as well as across several species (Schweiger and Linial, 2010). P-sites, especially phosphorylated serines (pS) and threonines (pT), and to a lesser extent tyrosines (pY), were shown to cluster more than expected by chance, which included accounting for the background distribution of serines, threonines, and tyrosines as well as taking into account the reported tendency of P-sites to preferentially occur in disordered regions (Dunker et al., 2002; Iakoucheva et al., 2004; Schweiger and Linial, 2010).

For the computational prediction of individual P-sites from protein sequence information alone, several software programs have been developed and are broadly available (for review, see Trost and Kusalik, 2011). Predicting P-hotspots in proteins then appears to simply be possible by choosing a preferred P-site prediction tool and then identifying clusterings of predicted sites along the sequence. However, here we show that this approach is hampered by the dense clustering of P-sites in hotspot themselves. When applied to the *Arabidopsis* genome, we demonstrate here that the *Arabidopsis*-trained P-site prediction method integrated in the PhosPhAt database cannot faithfully reproduce the actually observed distance relationships between consecutive experimentally determined P-sites. As the distance between consecutive P-sites is very short, 54% of all pS and pT sites are within four amino acids from another P-site (Schweiger and Linial, 2010), extracting a sequence motif for single and isolated sites is compromised by neighboring P-sites falling into the sequence window used in the prediction leading to poorly defined compositional profiles in the flanking sequences. For example, assuming that there may exist P-site tandems (pairs of sequence consecutive P-sites), for the N-terminal P-site, a C-terminal neighboring P-site (i.e., serines, threonines, or tyrosines) will be extracted, but for the second, C-terminal P-site an N-terminal position will appear enriched. Taken together, no clear sequence profile will be discernible.

Here, we analyzed the largest available set of experimentally detected P-sites in *A. thaliana* from the perspective of P-site clustering along the sequence, thereby confirming findings reported in earlier studies using different datasets (Schweiger and Linial, 2010). We furthermore investigated the spacing characteristics considering both the limited experimental coverage of protein sequence regions as well as the underlying spacing tendencies of serine, threonine, and tyrosine amino acid residues themselves.

Motivated by the shortcomings of predictors of individual P-sites to faithfully identify P-hotspots, we present a novel approach to the prediction of P-hotspots based on the amino acid sequence of proteins alone. As, because of the variable spacing between consecutive P-sites, no characteristic P-hotspot sequence motif was detectable that would permit the use of associated prediction approaches such as Hidden Markov Models (HMMs), we used the amino acid composition of P-hotspots as the primary feature extracted from protein sequences combined subsequently with Support Vector Machine (SVM) based classification methods and show that P-hotspots can be predicted with reasonable accuracy. We applied our developed P-hotspot prediction method, named HotSPotter, to the *Arabidopsis* proteome and identified candidate hotspots in several thousands of proteins. Combined with their functional characterization via annotation information, the potentially high abundance of P-hotspots sheds further light on their significance in regulatory processes and offers new avenues for the experimental study of P-hotspot phenomena.

All P-hotspot annotations, both experimentally annotated and computationally predicted sites, have been made available as part of the PhosPhAt database at http://phosphat.mpimp-golm.mpg.de (Heazlewood et al., 2008; Durek et al., 2010).

## Materials and Methods

### Mass-spectral peptides/phosphopeptides and experimentally determined P-sites/*Arabidopsis* proteome

Experimentally determined P-sites were obtained from the PhosPhAt database (Heazlewood et al., 2008; Durek et al., 2010) with data as of March 2010. At the time of this study, PhosPhAt contained information about 61,445 peptide spectra obtained from *Arabidopsis* samples that map to 10,507 *Arabidopsis* proteins. Filtering the dataset for unambiguously assigned P-sites in proteolytic peptides resulted in a dataset of 7,214 phosphopeptides mapping to 4,326 *Arabidopsis* proteins with protein sequences obtained from TAIR, version 9 (Huala et al., 2001). This dataset included 5,238 serine (73%), 1,433 threonine (20%), and 543 tyrosine (7%) P-sites, respectively. For 2,940 of the total of 7,214 experimentally verified P-sites, no other P-site was reported in the same protein. Thus, they have been excluded from further analysis leaving 4,274 P-sites for further studies on pairwise P-site distances.

### Inter-P-site distances

Sequence distances between detected P-sites, *d_i,j_* = *P_i_* − *P_j_*, with *P_i_* and *P_j_* corresponding to the respective sequence positions of two neighboring P-sites *i* and *j* were included in the analysis of their statistical distribution only if both sites mapped to a protein sequence segment that was continuously covered by experimentally measured peptides. Thus, potentially wrong distances due to incomplete peptide coverage were avoided as best as possible. For obtaining the statistics of inter-P-site distances, the respective closest P-site (the closest of either the next P-site in N-terminal or C-terminal sequence direction) was chosen, designated as *d*_N_.

### Randomizations of P-site distributions

The actual distance distribution between neighboring P-sites was compared to two sets of randomized P-site distributions. (a) P-flag-randomization: phosphorylation flags, i.e., detected phosphorylations of serine (S), threonine (T), or tyrosine (Y), were randomly reassigned to other S’s, T’s, or Y’s along the protein sequence or region with continuous peptide coverage. (b) Sequence-randomization: the entire protein sequence (or region with continuous peptide coverage) was randomized by randomly shuffling all amino acid residue positions. Method (a) tests whether the actually observed distances are merely a consequence of the underlying S, T, Y distributions, and their positional preferences. Method (b) destroys also these potential biases.

### Predicted P-sites in the *Arabidopsis* proteome

Computational predictions of individual P-sites were derived from the PhosPhAt P-site predictor (Durek et al., 2010). Only predictions resulting in a score >1 corresponding to high-confidence predictions were included in the analysis yielding 92,872 predicted P-sites associated with S, T, or Y in the *Arabidopsis* proteome. Thus, approximately 4% of all STY sites in the *Arabidopsis* proteome are predicted to be phosphorylated. Randomizations of predicted P-sites were created as described above for experimental sites.

### P-hotspots in the *Arabidopsis* proteome

#### Definition of P-hotspots

Sequence regions with occurrences of a least four consecutive experimentally detected P-sites along a protein sequence with a distance between neighboring sites of no more than 10 amino acids were considered as P-hotspots. The minimal hotspot length was set as 17 amino acid residues in order to provide a large enough sequence from which to derive a somewhat meaningful composition vector. Thus, sequence windows were padded by additional amino acid residues from the actual protein sequence beyond the terminal pSTY positions to result in hotspots of length 17, and minimally three residues on either side regardless of hotspot length.

### Prediction of P-hotspots using SVMs

#### Feature vectors, positive/negative training vectors

The prediction of P-hotspots was based on the amino acid composition of P-hotspots expressed as 420-dimensional vectors of the counts of occurrences of all 20 amino acids (A, …, Y) and all 400 possible amino acid dimers (AA, AC, …, YW, YY), both normalized by the considered P-hotspot sequence window length. N-to-C-terminal sequence directionality was considered in the amino acid pair counts such that amino acids were paired up only with their respective C-terminal amino acid residues in the same hotspot sequence resulting in asymmetric pair counts; i.e., some residual sequence order information is included in the prediction method.

The 79 P-hotspot sequences detected using experimentally identified P-sites ranging in length between 17 and 71 amino acid residues were subdivided into all possible segments of length 17 at a one amino acid increment resulting in 365 different segments and associated composition vectors representing the positive examples for the classification task. Thus, the considered sequence windows overlapped. For example, two neighboring sequence windows are identical in at least 16 positions. To account for this redundancy, in the training and testing the available P-hotspot sets were subdivided based on the P-hotspots. Identified P-hotspot sequences showed low mutual sequence identity with a median (maximal) pairwise sequence identity of 21.4% (50%) and no matching sequence segment longer than seven amino acid residues between any two hotspot sequences as determined from running an all-against-all sequence alignment using the program *align0* (Myers and Miller, 1988). Lastly, it was checked that indeed all sequences and associated feature vectors were non-redundant.

Proteolytic peptide-covered *Arabidopsis* protein sequences that are not identified as P-hotspots served as negative examples. As in the case for P-hotspots (positive examples), the corresponding consecutive sequence regions were subdivided into windows of length 17 and the 420-dimensional composition vectors were determined. Only those windows with serine, threonine, and tyrosine frequencies, respectively, as high as or greater than the lower limit determined for P-hotspots were selected as negative examples. This requirement was introduced to safeguard against trivial compositional differences resulting in increased phosphorylation rates as obviously fewer phosphorylatable amino acid residues will correlate with fewer P-sites. Furthermore, the risk of rather detecting unstructured regions as opposed to P-hotspots specifically is minimized. It is important to note that the negative set may contain individual P-sites. However, they were not sufficiently clustered to result in a P-hotspot assignment.

As the maximum negative set is substantially larger than the positive set, which would potentially bias the prediction toward specifically recognizing features associated with the negative rather with the positive set, the negative set was downsized to a ratio of 1:8 positive vs. negative examples by random selection.

#### SVM-based classification

The SVM-Light software package (http://svmlight.joachims.org/) was used as the classification engine. The radial basis function was used as the kernel. The associated parameters γ (kernel parameter), and the margin parameters *C* (the tolerance with regard to classification errors) and *j* (weight to balance errors of positive and negative examples) were identified by an exhaustive grid search implemented as a fivefold cross-validation based on maximal *F*-scores (see below). Intervals and increments were chosen as follows: *C* = (2^−10^, 2^−9^, 2^−8^, …, 2^24^), γ = (2^−10^, 2^−9^, 2^−8^, …, 2^6^), and *j* = (2^−2^, 2^−1^, 2^−0^, …, 2^4^), thus covering large intervals for all parameters. Best performance was achieved for *j* = 2^3^, *C* = 2^0^, *g* = 2^2^. Furthermore, the cost associated with false positive relative to false negative predictions was set to a ratio of 8:1 reflecting the difference in abundance in the training dataset. The performance results of the SVM-based P-hotspot predictions (named “HotSPotter”) were obtained in a fivefold cross-validation such that an average of the performance metric was obtained from five repeats of training/testing set splits of 80/20%. SVMs were trained on the 80%-set and performance parameters obtained from applying them to the 20% hold-out set. Here, the 79 original hotspot sequences were partitioned, and subsequently, the corresponding feature vectors were computed. As the hotspot sequences were largely dissimilar (see above), the performance assessment was effectively protected against overfitting. For the final application of HotSPotter to the *Arabidopsis* proteome with all protein sequences obtained from TAIR, version 9 (Huala et al., 2001), a final SVM was trained based on all 79 hotspots detected based on experimentally detected P-sites and applied to the *Arabidopsis* proteome in order to detect additional phosphorylation hotspots. Predictions were considered positive; i.e., a sequence window of length 17 was considered a P-hotspot segment, if the obtained SVM score was greater than initially 0, and subsequently when applied to the entire proteome, >1 to reduce the number of false positive predictions.

#### Post-prediction filtering and consolidation

Initially, all 12,866,960 possible overlapping 17-mers of the *Arabidopsis* proteome (33,410 proteins, TAIR-9; Huala et al., 2001) were subjected to the SVM classification. Subsequently, consecutive runs of positive predictions were consolidated into runs and filtered as follows. Every run consisting of consecutive positive predictions was tested to contain at least four S, T, or Y amino acid residues with the largest sequence spacing between them of no more than 10 amino acid residues; i.e., the same criteria as imposed on the experimental hotspots were applied. As prediction windows overlapped, predictions could potentially be conflicting for one and the same sequence position. For example, a 17-mer was predicted with positive score, while the adjacent 17-mer was predicted negative leading to conflicting assignments for 16 residue positions. In this case, the positive predictions took precedence. Furthermore, neighboring runs with fewer than or equal to 17 (window length) residues between their respective start positions were merged into a single run as they overlapped, but were interrupted by negative predictions.

### GO term enrichment analysis

Enrichment analysis of gene ontology (GO) terms in proteins observed and predicted to harbor P-hotspots was performed using the as described in Walther et al. (2007) applying a Fisher exact test with subsequent Benjamini–Hochberg correction (Benjamini and Hochberg, 1995) and using detailed GO and GO-slim term annotations as available from TAIR (Huala et al., 2001).

Tests were performed separately for the function, process, and component GO term categories. The entire *Arabidopsis* proteome minus the set of proteins to be tested and associated GO term annotations served as the respective reference sets.

### Prediction of disordered regions in *Arabiodpsis* proteins

Disordered regions in all 33,410 TAIR-9 *Arabidopsis* proteins were predicted using the program GlobPlot (Linding et al., 2003). GlobPlot was executed locally using the Globe Pipe script downloaded from http://globplot.embl.de/ and applying the recommended default parameters (webserver settings): SmoothFrame = 10, DOMjoinFrame = 15, DOMpeakFrame = 74, DISjoinFrame = 4, and DISpeakFrame = 5. Of all predicted disordered regions, only the 42,517 disordered regions of length 17 or greater were considered for further analyses to match the minimal length of predicted hotspot regions.

### Overlap of P-hotspots and disordered regions, randomization of P-hotspot positions in the *Arabidopsis* proteome

P-hotspots and disordered regions were considered overlapping if at least three consecutive amino acid residues were shared between them. To compare the actual overlap of predicted disordered regions and P-hotspots with random expectations, all 13,677 HotSPotter-predicted P-hotspots (SVM score >1) were reassigned to random positions in the *Arabidopsis* proteome by randomly choosing for all hotpots an *Arabidopsis* protein and then positioning the hotspot to a random position along the protein’s sequence and disallowing any hotspot overlaps. This process was repeated 10 times to obtain an average statistic.

## Results

### Distribution of P-sites in the *Arabidopsis* proteome

**Figure 1:**
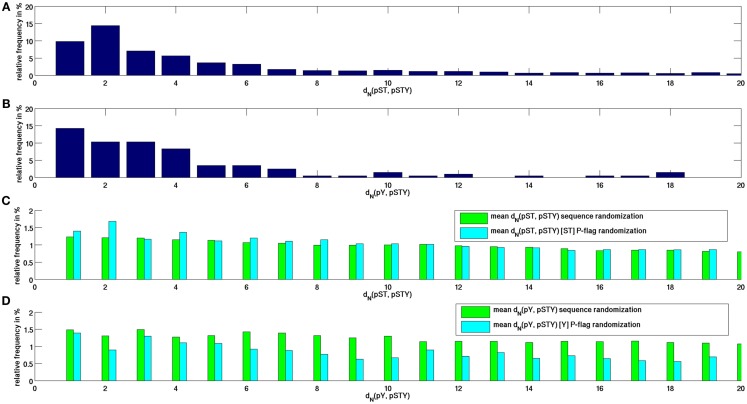
**Frequency distribution of sequence distances between neighboring P-sites between (A) any pST and any other site P-site pSTY, (B) any pY and any other P-site**. As for the respective neighboring site, no distinction was made as to what amino acid residue type (either S, T, or Y) was found phosphorylated. **(C)** Equivalent distributions for P-flag-randomized and sequence-randomized protein sequences averaged over 100 repeat runs for nearest pST and pSTY distances. **(D)** Similary for pY, pSTY distances. In P-flag, phosphorylation signals were randomly redistributed among the existing serines and tyrosines, whereas in sequence-randomized runs, the entire protein sequence was randomized.

We first characterized the sequence distances, *d*_N_, between P-sites to their respective closest neighboring P-site along the protein sequence as observed in peptide-covered regions in the *Arabidopsis* proteome. Because the obtained distance distributions for serine and threonine P-sites were found to be very similar, we treated them as a single event. As shown in Figure 1A and confirming earlier findings reported for a larger set of 51,000 P-sites from multiple organisms (Schweiger and Linial, 2010), we found that for phosphorylated serine and threonine residues and their distances to respective closest neighboring P-sites, there is a pronounced peak at distance *d*_N_ = 2 with an overall decreasing frequency of pair distances with increasing sequence separation. Note that the decreasing frequency with increasing sequence separation is to be expected as the probability that a sequence region is not phosphorylated – qualifying it as a spacing sequence between two neighboring P-sites – also falls. In contrast, the distribution of sequence distances of pYs (Figure 1B) to any other P-site was clearly different (*p* = 0.0015 in a chi-squared test for homogeneity comparing *d*(pY, pSTY) and *d*(pST, pSTY), exhibiting a monotonically decreasing frequency for increasing sequence separation. Compared to randomized protein sequences (Figure 1C), the enrichment of close sequence positions of neighboring P-sites of actual P-sites becomes evident. While for real P-sites, about 10% of all P-neighbor distances are at *d*_N_ = 1 and close to 50% of pairwise distances are found within *d*_N_ < 6, the equivalent number is only about 1% for randomized sequences and only about 6% of all distance intervals at *d*_N_ < 6. Thus, P-sites tend to cluster also when considering MS-peptide-covered sequence regions only.

Evidently, the distribution of P-sites along protein sequences is strongly influenced by the distribution of phosphorylate-able amino acid residue types themselves. We performed two different types of randomizations – one with randomly reassigning experimentally confirmed P-events to the real und unchanged positions of phosphorylate-able amino acid types, termed P-flag randomization, and another, termed sequence-randomization, in which the whole protein sequence was shuffled, thus destroying also any preferences of pairwise distances of phosphorylate-able amino acid types. Comparing both randomization approaches allows to better assess the influence of the underlying serine and threonine distributions on the observed actual distance distributions. Interestingly, the peak at interval *d*_N_ = 2 is also evident in the P-flag randomization runs, while the decay in relative frequency is monotonic if the whole protein sequence is randomized. Thus, it can be concluded that the observed preference for the sequence interval *d*_N_ = 2 for neighboring P-sites appears to be largely a consequence of the distribution of the serines and threonines themselves favoring distance intervals 2.

### Characteristics of actual P-neighbor distance distributions is not faithfully reproduced by predicted P-sites

The PhosPhAt database (Heazlewood et al., 2008; Durek et al., 2010) includes a set of computationally predicted P-sites using a prediction algorithm that was specifically trained using experimentally detected P-sites in the *Arabidopsis* proteome.

**Figure 2:**
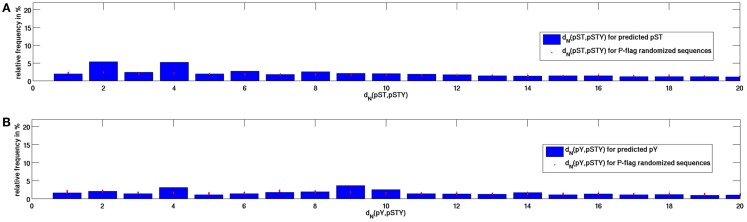
**Frequency distribution of distances between closest neighboring predicted P-sites in the *Arabidopsis* genome**. **(A)** Between any pST and the nearest pSTY, and **(B)** between any pY and the nearest pSTY. For comparison, results for 100 P-flag randomizations are given by red filled circles. Evidently, the nearest neighbor distance distribution differs from the distribution between experimentally identified sites (Figure 1) with a secondary peak at *d*_N_(pST, pSTY) = 4 and a more even distribution of *d*_N_(pY, pSTY) for predicted sites.

As shown in Figure 2, the frequency distribution between neighboring predicted P-sites differs substantially from the corresponding distribution observed for experimentally detected P-sites (Figure 1). Computational sites result in shallower distributions at close distances (only about 3% at *d*_N_ = 1 compared to >10% for experimental sites and 12% of all distances at *d*_N_ < 6 compared to close to 50% for real sites) and they exhibit another peak of locally preferred distances at *d*_N_ = 4 not observed for experimental sites. The mean distance between respectively closest neighboring P-sites was determined at 94.1 for experimental sites and 63.5 for predicted sites. As there are many more predicted (93,972) than experimental sites (7,214), the lower mean distance between neighboring predicted sites is to be expected. However, in P-flag randomizations, the mean distance increased for experimental sites to 142.7, whereas it increases only marginally to 67.9 for predicted sites even though only about 4% of all STYs in the *Arabidopsis* proteome are predicted to be phosphorylated such that any clustering of actual prediction sites could be destroyed in P-flag randomizations. Thus, compared to random background, experimental sites exhibit a much greater tendency to cluster than predicted P-sites.

We conclude that the approach of using computationally predicted P-sites for the purpose of identifying P-hotspots in the *Arabidopsis* proteome is bound to produce less than optimal results as it does not faithfully predict the characteristics of P-site distance distributions and the tendency to cluster in sequence. Therefore, here we pursued an alternative approach that relies more on compositional preferences of a sequence segment rather than a sequence position-specific amino acid profile as commonly used in computational P-site prediction algorithms.

### P-hotspots in the *Arabidopsis* proteome

First, we identified P-hotspots based on experimentally detected P-sites in the *Arabidopsis* proteome. Sequence regions with occurrences of a least four consecutive experimentally detected P-sites along a protein sequence with a distance between neighboring sites of no more than 10 amino acids were considered P-hotspots. The longest such P-hotspot was detected in a serine/arginine-rich protein splicing factor protein (AT2G37340.1) with 12 P-sites along a sequence region comprising 64 out of the total of 290 amino acid residues in that protein. In total, 79 P-hotspots were detected in 75 *Arabidopsis* proteins based on experimental P-sites (Table 1). As described similarly by Riano-Pachon et al. (2010) using a P-hotspot definition based on comparisons to background distributions, P-hotspot proteins identified in this study were found to be involved primarily in RNA binding and splicing processes (GO term enrichment analysis yielding *p*_FDR_ = 0.001 for GO function term “RNA binding,” *p*_FDR_ = 4.2E−13 for GO process term “RNA splicing”), and are located in the “nuclear speck” (*p*_FDR_ = 1.3E−09), the “plasma membrane” (*p*_FDR_ = 4.7E−06), and the “splicosome” (*p*_FDR_ = 0.05).

**Table 1:** ***Arabidopsis* P-hotspot containing proteins detected based on experimentally identified phosphorylation sites**.

AGI ID	Gene symbol	Annotation	Phosphorylation sites	Start of hotspot	Hotspot sequence
AT1G01540.1		Protein kinase family protein	102, 106, 107, 110	98	RVVFSDRVSSGESRGTA
AT1G07985.1		Expressed protein	130, 132, 134, 138	126	KVVGSSSPTNIHSKSWR
AT1G08680.1	ZIGA4, AGD14	ZIGA4 (ARF GAP-like zinc finger-containing protein ZiGA4); ARF GTPase activator/DNA binding/zinc ion binding	190, 191, 194, 195	184	GLHAKASSFVYSPGRFS
AT1G20440.1	COR47, RD17	COR47 (COLD-REGULATED 47)	89, 98, 108, 113	86	QEKTEEDEENKPSVIEKLHRSNSSSSSSSDE
AT1G26540.1		Agenet domain-containing protein	324, 328, 334, 336	321	HLRSFLNSKEISETPTKAK
AT1G27500.1		Kinesin light chain-related	32, 33, 36, 44	29	ELQSSNQSPSRQSFGSYGD
AT1G29220.1		Transcriptional regulator family protein	80, 82, 86, 89	76	GVGASSSAHGTPRSLDN
AT1G29350.1		Expressed in: male gametophyte, guard cell, pollen tube; expressed during: L mature pollen stage, M germinated pollen stage; BEST *Arabidopsis thaliana* protein match is: kinase-related (TAIR:AT1G29370.1)	105, 107, 108, 112	100	RYAGRSGSTHFSSTDSG
AT1G35580.1	CINV1	CINV1 (cytosolic invertase 1); beta-fructofuranosidase	43, 45, 48, 49	38	SFDERSMSELSTGYSRH
AT1G35580.1	CINV1	CINV1 (cytosolic invertase 1); beta-fructofuranosidase	60, 65, 69, 72, 73	57	IHDSPRGRSVLDTPLSSARN
AT1G45688.1		Unknown protein	15, 19, 29, 31, 34	12	AASSPARSPRRPVYYVQSPSRDSHDG
AT1G55310.1	SR33, ATSCL33, SCL33	SR33; RNA binding/proteinbinding	4, 5, 6, 8	0	MRGRSYTPSPPRGYGRR
AT1G59710.1		Expressed in: 23 plant structures; expressed during: 13 growth stages; contains InterPro domain/s: protein of unknown function DUF569 (InterPro:IPR007679), actin_cross-linking (InterPro:IPR008999)	195, 196, 198, 203	191	FRQESTDSLAVGSPPKS
AT1G59870.1	PEN3, PDR8, ATPDR8	PEN3 (penetration 3); ATPase, coupled to transmembrane movement of substances/cadmium ion transmembrane transporter	36, 39, 42, 44	32	EDIFSSGSRRTQSVNDD
AT1G62830.1	LDL1, SWP1, ATSWP1	LDL1 (LSD1-LIKE1); amine oxidase/electron carrier/oxidoreductase	820, 822, 830, 831	817	ERKSLSQEGESMISSLKA
AT1G66680.1	AR401	AR401	34, 44, 46, 53	31	SLASDDDRSIAADSWSIKSEYGSTLD
AT1G73200.1		Phosphoinositide binding	312, 314, 316, 317	306	VQVISRSWSHSSHASDV
AT1G76920.1		F-box family protein (FBX3)	177, 178, 180, 190	174	ALYYSGTVVANQWLKFSSNL
AT1G80530.1		Nodulin family protein	270, 271, 275, 276	265	RSNAKSSPLGSSDNLAK
AT2G01190.1		Octicosapeptide/Phox/Bem1p (PB1) domain-containing protein	381, 386, 394, 399	378	RVYSDDERSDHGVQAGYRKPPTPRS
AT2G23350.1	PAB4, PABP4	PAB4 [POLY(A) binding protein 4]; RNA binding/translation initiation factor	640, 647, 649, 656	637	SQGSEGNKSGSPSDLLASLSIND
AT2G26730.1		Leucine-rich repeat transmembrane protein kinase, putative	631, 632, 636, 639, 648	628	LRQSSDDPSKGSEGQTPPGESRTP
AT2G29210.1		Splicing factor PWI domain-containing protein	390, 392, 395, 400, 402, 405	387	RRRSPSPLYRRNRSPSPLYRRN
AT2G31650.1	ATX1, SDG27	ATX1 (*Arabidopsis* HOMOLOG of trithorax); histone-lysine N-methyltransferase/phosphatidylinositol-5-phosphate binding	481, 482, 484, 487, 489	477	MRKFTSLTDHSASALYK
AT2G35030.1		Pentatricopeptide (PPR) repeat-containing protein	110, 112, 116, 121	107	NVVTWTAMVSGYLRSKQL
AT2G35350.1	PLL1	PLL1 (poltergeist like 1); catalytic/protein serine/threonine phosphatase	188, 190, 192, 198	185	GEISRSNSAGVHFSAPL
AT2G35880.1		Expressed in: 24 plant structures; expressed during: 13 growth stages; contains InterPro DOMAIN/s: targeting for Xklp2 (InterPro:IPR009675)	108, 111, 116, 117	104	YTDITRKSIDATTSKTS
AT2G37340.1	RSZ33, ATRSZ33	RSZ33; nucleic acid binding/nucleotide binding/zinc ion binding	201, 203, 210, 218, 225, 229, 238, 244, 252, 255, 264, 265	198	MDDSLSPRARDRSPVLDDEGSPKIIDGSPPPSPKLQKEVGSDRDGGSPQDNGRNSVVSPVVGAGGDSSKED
AT2G41705.1		Camphor resistance CrcB family protein	60, 64, 65, 68	56	RRRHSAGRSSRLSADDF
AT2G41720.1	EMB2654	EMB2654 (EMBRYO DEFECTIVE 2654)	529, 531, 537, 539	526	KADSVTFTILISGSCRM
AT2G41740.1	VLN2, ATVLN2	VLN2 (VILLIN 2); actin binding	845, 848, 854, 855	842	NKKSPDTSPTRRSTSSN
AT2G43680.1	IQD14	IQD14; calmodulin-binding	125, 127, 132, 142	122	VPRTLSPKPPSPRAEVPRSLSPKP
AT2G45540.1		WD-40 repeat family protein/beige-related	1612, 1613, 1618, 1621	1608	SSERSSGNSVTLDSGSQ
AT2G46170.1		Reticulon family protein (RTNLB5)	27, 28, 29, 30, 31	21	KIHHHDSSSSSESEYEK
AT2G46495.1		Zinc finger (C3HC4-type RING finger) family protein	401, 405, 407, 410	397	KRLLTFNISGSPFSPRF
AT3G05090.1		Transducin family protein/WD-40 repeat family protein	368, 378, 385, 392	365	EVQSPKTVFQRGGSFLAGNLSFNRARVSLEG
AT3G07790.1		DGCR14-related	117, 120, 121, 127	114	KTQTPGSTFLRNFTPLD
AT3G13570.1	SCL30A	SCL30a; RNA binding/nucleic acid binding/nucleotide binding	165, 173, 175, 177	162	GYNSPPAKRHQSRSVSPQD
AT3G13990.1		Unknown protein	493, 495, 498, 501	489	RVSRSDSPVSAVSEPQL
AT3G17420.1	GPK1	GPK1; ATP binding/kinase/protein kinase/protein serine/threonine kinase	58, 62, 69, 74, 75	55	VTQSPRFTEEIKEISVDHGSSNNN
AT3G23100.1	XRCC4	XRCC4; protein C-terminus binding	224, 225, 230, 233	220	EEEESTDKAESFESGRS
AT3G27960.1		Kinesin light chain-related	573, 577, 581, 582, 588	570	CGPYHPDTLAVYSNLAGTYDAM
AT3G29310.1		Calmodulin-binding protein-related	324, 325, 326, 331	319	NRHDLTSSAEDDSVDGD
AT3G29390.1	RIK	RIK (RS2-interacting KH protein); RNA binding	511, 513, 516, 517	506	PPRSKTMSPLSSKSMLP
AT3G48530.1	KING1	KING1 (SNF1-related protein kinase regulatory subunit gamma 1)	11, 13, 18, 21, 22	8	IMRSESLGHRSDVSSPEA
AT3G52400.1	SYP122, ATSYP122	SYP122 (syntaxin of plants 122); SNAP receptor	7, 17, 21, 27	4	LSGSFKTSVADGSSPPHSHNIEMSKAK
AT3G52930.1		Fructose-bisphosphatealdolase, putative	31, 32, 34, 41	28	ADESTGTIGKRLASINV
AT3G53500.1	RSZ32	Zinc knuckle (CCHC-type) family protein	172, 174, 183, 188, 190, 193	169	RDQSLSPDRKVIDASPKRGSDYDGSPKE
AT3G55460.1	SCL30	SCL30; RNA binding/nucleic acid binding/nucleotide binding	3, 4, 7, 9	0	MRRYSPPYYSPPRRGYG
AT3G55460.1	SCL30	SCL30; RNA binding/nucleic acid binding/nucleotide binding	175, 177, 179, 181	170	DSRSRYRSRSYSPAPRR
AT3G55460.1	SCL30	SCL30; RNA binding/nucleic acid binding/nucleotide binding	203, 204, 205, 208	197	ENYSRRSYSPGYEGAAA
AT3G56510.1		TBP-binding protein, putative	232, 237, 238, 240	228	RQKKSIENETSQSKPGL
AT3G58940.1		F-box family protein	112, 115, 118, 121	108	QRGVSDLYLFTDFSDED
AT3G61860.1	ATRSP31, RSP31	RSP31; RNA binding/nucleic acid binding/nucleotide binding	246, 252, 254, 256	243	RQRSPGYDRYRSRSPVP
AT3G62280.1		Carboxylesterase/hydrolase, acting on ester bonds	90, 93, 95, 98, 100	87	LKMTYLSPYLDSLSPNF
AT4G05150.1		Octicosapeptide/Phox/Bem1p (PB1) domain-containing protein	263, 265, 269, 276	260	EVSTLSDPGSPRRDVPSPYG
AT4G07523.1		Transposable element gene; similar to unknown protein (*Arabidopsis thaliana*) (TAIR:AT5G27180.1)	3, 5, 6, 8, 9, 10		MPLSYSSPSSSEERSDD
AT4G11740.1	SAY1	SAY1	312, 314, 323, 325, 327	309	RAASGSLAPPNADRSRSGSPEE
AT4G13510.1	AMT1;1, ATAMT1, ATAMT1;1	AMT1;1 (AMMONIUM TRANSPORTER 1;1); ammoniumtransmembranetransporter	487, 489, 491, 495	483	VEPRSPSPSGANTTPTP
AT4G25160.1		Protein kinase family protein	312, 314, 321, 323	309	TRFSWSGMGVDTTHSRAS
AT4G25580.1		Stress-responsive protein-related	155, 157, 161, 167	152	GAPTLTPHNTPVSLLSATE
AT4G31580.1	SRZ-22, SRZ-22, RSZP22	SRZ-22; protein binding	159, 169, 171, 173, 177	156	RRRSPSPPPARGRSYSRSPPPYRAR
AT4G31700.1	RPS6	RPS6 (ribosomal protein S6); structural constituent of ribosome	230, 236, 239, 240, 246	227	RSESLAKKRSRLSSAAAKPSVTA
AT4G32250.1		Protein kinase family protein	12, 21, 23, 29, 30	9	PDDTEYEIIEGESESALAAGTSPWM
AT4G35785.1		Nucleic acid binding/nucleotide binding	40, 42, 48, 50	37	RSRSRSLPRPVSPSRSR
AT4G38600.1	KAK, UPL3	KAK (KAKTUS); ubiquitin-protein ligase	1366, 1367, 1372, 1373, 1374	1362	EGKITSLDDLSTTAAKV
AT4G39680.1		SAP domain-containing protein	310, 318, 319, 323	307	AGDSEKLNLDRSSGDESMED
AT5G02240.1		Binding/catalytic/coenzyme binding	234, 235, 236, 238	228	GSKPEGTSTPTKDFKAL
AT5G04930.1	ALA1	ALA1 (aminophospholipid ATPase1); ATPase, coupled to transmembrane movement of ions, phosphorylative mechanism	39, 46, 51, 57	36	DLGSKRIRHGSAGADSEMLSMSQKE
AT5G06210.1		RNA binding protein, putative	136, 138, 139, 141, 142	129	DPAVIAATRTTETSKSD
AT5G18660.1	PCB2	PCB2 (Pale-green and chlorophyll B reduced 2); 3,8-divinyl protochlorophyllide a 8-vinyl reductase	370, 378, 381, 382	367	AAESMLILDPETGEYSEEK
AT5G21160.1		La domain-containing protein/proline-rich family protein	369, 377, 380, 383	366	SAETIGDGDKDSPKSITSGDN
AT5G41600.1	BTI3	BTI3 (VIRB2-interacting protein 3)	24, 25, 28, 30	19	HGHGDSSSLSDSDDDKK
AT5G47690.1		Binding	1273, 1280, 1283, 1288	1270	HLESDMDKNVSLDSHDENSDQE
AT5G52040.1	ATRSP41	ATRSP41; RNA binding/nucleic acid binding/nucleotide binding	191, 201, 209, 218, 219, 228, 230, 232, 238	188	RRRSPSPYRRERGSPDYGRGASPVAHKRERTSPDYGRGRRSPSPYKRARLSPDY
AT5G52040.1	ATRSP41	ATRSP41; RNA binding/nucleic acid binding/nucleotide binding	336, 341, 346, 348, 350	333	GRGYDGADSPIRESPSRSPPA
AT5G57110.1	ACA8, AT-ACA8	ACA8 (autoinhibited Ca2^+^-ATPASE, isoform 8); calcium-transporting ATPase/calmodulin-binding/protein self-association	18, 21, 26, 28	15	DVESGKSEHADSDSDTF
AT5G62820.1		Integral membrane protein, putative	27, 30, 40, 46	24	RFHSPLSDAGDLPESRYVSPEGSPFK
AT5G64200.1	ATSC35, SC35	ATSC35; RNA binding/nucleic acid binding/nucleotide binding	273, 277, 279, 282	269	PERRSNERSPSPGSPAP

### P-hotspot predictions

#### Performance of the SVM-based P-hotspot classification algorithm termed HotSPotter

Using the detected hotspots for training, we developed a SVM based computational algorithm, termed HotSPotter, to predict additional hotspots in the *Arabidopsis* proteome as outlined in the Section “Materials and Methods.” Instead of sequence motifs that are conventionally used to predict individual P-sites, our method relies on the amino acid composition of P-hotspot regions. In a fivefold cross-validation setting, the following performance parameters were obtained (Table 2). When tuning the performance, we deliberately tolerated relatively low true positive rates (63% achieved), in exchange for a low false positive rate (2%). In training, the proportion of positive to negative examples was adjusted in favor of positive examples, and not the dominating negative signal (see Materials and Methods). When applied to the entire *Arabidopsis* genome, low false positive rates were deemed critical as the number of true negatives is much higher than in training.

**Table 2:** **HotSPotter prediction performance**.

		Actual		
		Positive (hotspot sequence)	Negative (non-hotspot sequence)	
Predicted	Positive	TP = 63% (230/365)	FP = 2% (51/2915)	PPV = 82%
	Negative	FN = 37% (135/365)	TN = 98% (2864/2915)	NPV = 95%

*Contingency table of prediction results of the SVM-based classification termed HotSPotter applied to sequence windows of length 17 and obtained in fivefold cross-validation*.

*TP, true positive; FP, false positive; FN, false negative; TN, true negative; PPV, positive predictive value; NPV, negative predictive value. Numbers in parentheses refer to the actual counts. Here, positive prediction results are based on SVM scores >0*.

#### P-hotspot predictions in the *Arabidopsis* genome using HotSPotter

We applied the trained SVM classification algorithm to the prediction of phosphorylation hotspots in the *Arabidopsis* genome. Applying the original SVM cutoff score value, *S*_SVM_, for positive predictions of *S*_SVM_ > 0 resulted in positive predictions for 7.3% of all tested sequence windows, length 17, and correspondingly, 23,525 (70%) of all *Arabidopsis* proteins. Despite the low false positive rate obtained in cross-validation of only 2% during training, this number seems very high. Therefore, we applied a more stringent cutoff of *S*_SVM_ > 1, reducing the number of predicted hotspot proteins to 9,599 (28%) of all *Arabidopsis* proteins including 16 mitochondrial and 12 chromosomal proteins (Table 3).

**Table 3:** **Statistics of HotSPotter predictions in the *Arabidopsis* proteome**.

(A) SVM score threshold, S, for positive prediction	(B) Number of windows, length 17	(C) Number of windows with positive score	(D) Number of STY- content filtered and run-consolidated hotspots	(E) Number of unique proteins (genes) containing hotspots
S > 0	12,866,960	945,670 (7.3%)	54,329 (44,247)	23,524 (19,252)
S > 1		160,780 (1.2%)	13,677 (11,065)	9,599 (7,847)

*(D) Numbers refer to counts of hotspots after checking for STY contents (≥4 and no more than 10 residues between any STY) and merged consecutive runs of positive windows. Numbers in parentheses refer to unique hotspot sequences. (E) The number of proteins refers to all proteins derived from all genes including annotated splice forms*.

Of the 75 proteins observed to contain P-hotspots based on experimental data, 57 (76%) were contained in the predicted set of hotspot containing proteins based on positive predictions at SVM score, *S*_SVM_ > 1.

#### Biological role of hotspot proteins/GO term enrichment/depletion analysis

We performed a GO term enrichment analysis to characterize the proteins predicted to harbor P-hotspots with regard to their association to particular biological processes, functions, and components. Using GO-slim term annotations and probing both for enrichment as well as depletion of annotation terms in the set relative to all other *Arabidopsis* proteins, we found that hotspot proteins appear to be involved in DNA/RNA metabolism processes, as well as signal transduction (e.g., kinase and transcription factor functions) and developmental processes. With regard to location, predicted hotspot proteins appear to be targeted toward the chloroplast, nucleus (consistent with DNA/RNA binding functions), and associated with the plasma membrane. By contrast, as of yet uncharacterized *Arabidopsis* proteins as well as proteins involved in stress response, proteins performing transporter activities, and those with ribosomal or extracellular and cytosolic localization appear to be specifically depleted in the set of predicted P-hotspot proteins (Table 4).

**Table 4:** **Gene ontology-slim terms statistically enriched or depleted in the set of 9,599 *Arabidopsis* proteins (corresponding to 7,847 genes) predicted to contain P-hotspots based on SVM scores >1**.

FDR *p*-value	GO-slim process	FDR *p*-value	GO-slim function	FDR *p*-value	GO-slim component
**ENRICHMENT**					
9.27E−08	DNA or RNA metabolism	1.31E−28	Nucleotide binding	2.50E−67	Chloroplast
2.15E−07	Developmental processes	8.36E−16	Kinase activity	6.70E−16	Nucleus
2.38E−07	Cell organization and biogenesis	3.26E−15	Transcription factor activity	4.59E−13	Plasma membrane
1.38E−02	Other cellular processes	4.80E−14	Protein binding	1.22E−12	Plastid
1.52E−02	Signal transduction	4.67E−06	Transferase activity	3.00E−05	Other intracellular components
		1.09E−02	Hydrolase activity	4.82E−02	Golgi apparatus
		1.43E−02	DNA or RNA binding		
**DEPLETION**					
3.16E−05	Transport	6.83E−16	Unknown molecular functions	2.28E−48	Other cellular components
1.51E−04	Unknown biological processes	1.31E−15	Other binding	1.57E−19	Unknown cellular components
6.52E−03	Response to stress	4.11E−12	Other enzyme activity	1.97E−08	Extracellular
		6.50E−09	Structural molecule activity	2.93E−08	Cytosol
		4.43E−04	Nucleic acid binding	1.07E−07	Ribosome
		2.68E−03	Transporter activity	1.03E−02	Other membranes
		1.09E−02	Other molecular functions	1.10E−02	ER
				2.25E−02	Cell wall

*Removing the set of 57 proteins jointly contained in the experimental and predicted set as well as removing chloroplastidial and mitochondrial proteins did not result in any significant changes of GO-slim term enrichment/depletion statistics*.

#### P-hotspots and disordered regions

It is known that P-sites tend to occur in sequence regions linking protein structural domains (Riano-Pachon et al., 2010) and, generally, in unstructured regions defined as the absence of secondary structure or any other regular folding patterns with a correspondingly relatively high structural flexibility. Those regions are characterized by their own compositional preferences that may primarily be associated with their unstructuredness and to a lesser extend with their phosphorylation (Iakoucheva et al., 2004). In order to assess the co-occurrence of P-hotspots and disordered regions and to determine whether P-hotspots are associated with specific amino acid compositional preferences, we scanned all *Arabidopsis* proteins for disordered regions using the GlobPlot program (Linding et al., 2003). GlobPlot relies on the propensities of all 20 amino acids to occur in disordered regions obtained from an analysis of experimentally determined protein structure and computed running averages over sequence windows of given lengths. Thus, the approach resembles the HotSPotter method by also exploiting sequence compositional preferences. Any significant co-segregation suggesting redundancy of the underlying compositional preferences is thus best discernible.

Of all 13,677 HotSPotter-predicted P-hotspots (required SVM score >1), 5,285 hotspots (39%) overlapped with GlobPlot-predicted disordered regions (see Materials and Methods) in the respected *Arabidopsis* proteins. By comparison, on average only 2,152 ± 25 randomly positioned hotspots (16%) overlapped with disordered regions. Thus, a significant preference for P-hotspots to occur in disordered regions is evident. However, by far not all predicted disordered regions (5,554 out of a total of 42,517 disordered regions of length 17 or longer) are simultaneously predicted as P-hotspots (Note that the number of overlapping hotspots with disordered regions may differ from the respective number of overlapping disordered regions and hotspots. For example, a single hotspot may overlap with two sequence-separated disordered regions.)

While there is a significant correlation between unstructuredness and the likelihood of being a P-hotspot, the two sets are sufficiently disjoint to conclude that P-hotspots are associated with specific amino acid compositional preferences picked up on by the developed HotSPotter methodology.

#### Availability of P-hotspot annotations and HotSPotter predictions under PhosPhAt

**Figure 3:**
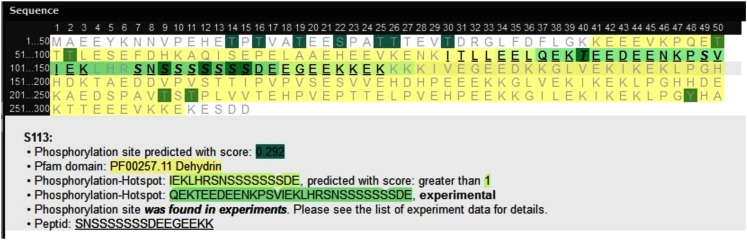
**Screenshot of the PhosPhAt database with P-hotspot annotation information**.

All hotspots based both on experimental P-sites as well as on predictions applying HotSPotter have been integrated into the PhosPhAt database and are available at http://phosphat.mpimp-golm.mpg.de. Figure 3 shows an example of a protein sequence containing both an experimental hotspot that was also predicted with SVM score >1. The HotSPotter predictions in the entire *Arabidopsis* proteome are available as Supplementary information of this publication (Table S1 in Supplementary Material) and under PhosPhat at http://phosphat.mpimp-golm.mpg.de/hotspots.html, where all training data are available as well.

## Discussion

The potential of modulating the function of proteins by surface charge effects mediated by locally enriched phosphorylation events has gained increased attention in recent years (Serber and Ferrell, 2007; Strickfaden et al., 2007). Besides position-specific single phosphorylation events associated with defined structural changes or modulated binding affinities of the carrier protein, local enriched clusterings of several P-sites that are themselves less restricted to precise locations along a protein sequence, may have evolved as an efficient and mutation tolerant mechanism to allow modulated molecular responses to changed external conditions.

Here, we firstly confirmed earlier reports (Schweiger and Linial, 2010) that indeed P-sites exhibit a tendency to cluster along a protein sequence in *A. thaliana*. By using experimental data and considering only peptide-covered regions, thereby excluding possible artifacts from differential coverage, we validated this trend with increased confidence. In this context, it may be worthwhile to investigate the influence of the three-dimensional protein structure on the clustering in the one-dimensional sequence. If only solvent exposed sites can actually be phosphorylated and assuming that the polypeptide chain folds such that solvent exposed and surface buried segments alternate, P-site clustering would directly follow from structure alone.

We showed that the characteristic distances of any serine or threonine to the next P-site differ significantly from the equivalent distribution for tyrosine sites (Figure 1). This difference is likely caused by the differences in target recognition of serine/threonine kinases compared to tyrosine kinases (Sugiyama et al., 2008). For example, CDK kinases phosphorylating serines and threonines have been reported to be associated with P-clusters (Moses et al., 2007). By comparing the actual intervals between neighboring P-sites to randomized phosphorylation events using the unchanged protein sequence (referred to as P-flag randomization), we concluded that the characteristic preference of sequence interval 2 for pST sites to their respective next neighboring P-site is likely caused by the underlying preference of serines and threonines themselves to occur at this spacing.

Based on experimentally identified P-sites in the *Arabidopsis* proteome, we detected 79 phosphorylation hotspots in 75 proteins. In this study, we set out to expand this set by computational predictions as it can be assumed that the actual set of P-hotspot in the *Arabidopsis* genome is much larger. The principal difficulty with all experimental approaches to detect phosphorylation events lies in the fact that actual P-sites may not be phosphorylated under the respective experimental conditions tested, but under different ones not yet known or tested. Computational approaches can be assumed to potentially overcome this issue by identifying characteristic molecular properties of clustered P-sites in general. The obvious strategy to first predict individual P-sites using an established and plant-specific P-site prediction program (Durek et al., 2010) was determined to lead to inaccurate results with resulting distance intervals between neighboring predicted P-sites differing significantly from the experimental ones (Figures 1 and 2). We believe that this may be caused by the details of the underlying prediction method that aims to exploit the position-specific flanking sequence around P-sites essentially translating them into motifs. However, if multiple P-sites are contained in the flanking sequence, the extracted sequence motif will be a mix of signals influenced also by the neighboring P-sites falling into the respective flanking region.

Inaccurate phosphorylation hotspots predictions based on individual predicted P-sites may also result from inaccuracies in the underlying experimental detection of clustered P-sites. In mass spectrometry, it is very difficult and often ambiguous to precisely interpret mass spectra of closely related phosphopeptides (MacLean et al., 2008). Particularly, if more than one of neighboring S/T/Y sites can be phosphorylated under a given condition, fragment mass spectra often result from different phosphorylated versions of the same peptide sequence.

An obvious idea for predicting hotspots may be to treat hotspots as a sort of structural domain and apply established recognition programs. We tested the HMMER program that is based on HMMs (Finn et al., 2011) for suitability of P-hotspot prediction. However, no satisfying alignment of the different hotspot sequences was possible [21% (50%) median (maximal) pairwise sequence identity as outlined in the see Materials and Methods], and thus, the obtained HMMs performed poorly and were dismissed. The failure to create robust alignments is certainly associated to the very characteristic of clustered sites that P-sites are locally enriched but without any apparent spacing constraints.

Thus, a method had to be devised that relies less on the position-specific sequence context and more on the properties of a region as a whole. For this purpose, we based our prediction efforts on the amino acid composition of hotspot sequence regions, thereby ignoring any precise sequence order information. Feeding amino acid composition vectors associated with P-hotspot and non-hotspot sequences into SVMs resulted in predictions of surprisingly high accuracy (Table 3) in a rigorous cross-validation setting.

In any computational classification tasks, overfitting; i.e., using too many features to describe the particular dataset at hand leading to poor generalizability of the classification algorithm by emphasizing the noise rather than the true relationship, is of great concern, especially when the available dataset size for training is small as is the case here with only 79 hotpot based on experimental data associated with 365 sequence windows of length 17. Despite using many features (420) and presenting them to the SVM classification engine, the imposed cross-validation protocol with very little sequence redundancy between any training and testing partition (see Materials and Methods) efficiently safeguarded against falsely obtaining good prediction results due to overfitting as generalizability is truly tested. Instead of using all amino acid types as separate variables, the number of features could be reduced by prior filtering of uninformative features or by combining several features into meta-features, e.g., amino acid types sharing similar physic-chemical properties.

The choice of features is one of the most crucial steps in any classification approach. With regard to P-hotspot prediction, there may exist better features and combinations thereof than those used here. For example, the feature set used in this study may even be augmented by adding additional, but orthogonal information such as secondary structural state or disorder score. However, given the discussed shortcomings of sequence motif based methods to reproduce the correct spacing between neighboring P-sites, our goal was to provide a proof of principle that methods that rely more on sequence-independent features such as composition can lead to reasonable results when applied to the prediction of P-hotspots.

Phosphorylation sites have been reported to occur in linker regions between structural domains and unstructured regions, in general (Iakoucheva et al., 2004; Riano-Pachon et al., 2010). To safeguard against falsely predicting unstructured regions rather than specifically P-hotspots, we used a set of negative examples with serine/threonine/tyrosine contents similar to positive hotspot examples. Furthermore, in training the SVM, we deliberately included regions as negative examples that also contained single, but isolated (no hotspot) P-sites. Nonetheless, if indeed there is a link between unstructuredness and the tendency of becoming phosphorylated, disentangling both contributions remains a challenging endeavor. While a significant overlap between unstructured regions and P-hotspot was evident, the two structural feature sets were still largely disjoint (61% of all predicted P-hotspots and 87% of all predicted disordered regions in the *Arabidopsis* proteome do *not* overlap with the respective other structural feature) leading us to conclude that the developed HotSPotter method does indeed specifically capture sequence compositional preferences associated with P-hotspots and not those of unstructured regions alone.

In light of the obtained classification results, we believe the obtained predictions for the whole *Arabidopsis* proteome may be sufficiently accurate to warrant additional studies on those proteins and their phosphorylation hotspots. The set of proteins predicted to harbor P-hotspots was found enriched in functions associated with signaling and binding events thus confirming the surmised role of P-hotspots as modulators of binding events and localization. We predict P-hotspots to be present in around 9,500 *Arabidopsis* proteins. Thus, P-hotspot regulation may be more frequent than previously acknowledged.

By making all P-hotspot predictions in *Arabidopsis thaliana* available we hope to offer to the scientific community a starting point for experimental verification and further study of phosphorylation hotspot mediated regulation processes.

## Conflict of Interest Statement

The authors declare that the research was conducted in the absence of any commercial or financial relationships that could be construed as a potential conflict of interest.

## Supplementary Material

The Supplementary Material for this article can be found online at http://www.frontiersin.org/Plant_Proteomics/10.3389/fpls.2012.00207/abstract

Supplementary Table S1 **Predicted hotspots in the *Arabidopsis* proteome based SVM score >1**. (Excel Table). File also contains data sheet of *Arabidopsis* P-hotspot containing proteins detected based on experimentally identified phosphorylation sites.
